# Difficulties of Online Physical Education Classes in Middle and High School and an Efficient Operation Plan to Address Them

**DOI:** 10.3390/ijerph17197279

**Published:** 2020-10-05

**Authors:** Hyun-Chul Jeong, Wi-Young So

**Affiliations:** 1 Department of Physical Education, Jeonbuk National University High School, Jeonju-si, Jeollabuk-do 54896, Korea; 01086445918@hanmail.net; 2 Sports and Health Care Major, College of Humanities and Arts, Korea National University of Transportation, Chungju-si 27469, Korea

**Keywords:** coronavirus disease-19 pandemic, online evaluation, online physical education class, teaching expertise in physical education, value of physical education

## Abstract

This study examined the difficulties of running online physical education classes in the context of coronavirus disease 2019 (COVID-19) and used the findings to develop an efficient operation plan to address these difficulties. Six middle and high school physical education teachers participated; three were experts in online physical education and active in the Korea Council School Physical Education Promotion, and three were recommended teachers making efforts to improve the online classes offered by the Korea Ministry of Education. A qualitative case study method employing phenomenological procedures to collect and analyze the data was used. The difficulties of operating middle and high school online physical education classes for the first time included (1) the monotony of the classes within their limited environmental conditions and limited educational content that did not adequately convey the value of physical education, (2) trial-and-error methods applied nationwide, resulting from a lack of expertise in operating online physical education classes, and (3) very limited evaluation guidelines proposed by the Korea Ministry of Education, which made systematic evaluation with online methods impossible. To address the identified problems and facilitate the efficient operation of online physical education classes, changes in strategic learning methods are needed to understand online physical education characteristics and thereby better communicate the value of physical education. It is also necessary to cultivate teaching expertise through sharing online physical education classes, where collaboration among physical education teachers is central. In addition, evaluation processes should be less formal to encourage active student participation.

## 1. Introduction

The entire world is currently facing a catastrophic situation resulting from the coronavirus disease 2019 (COVID-19) pandemic, which has affected the daily lives of people worldwide. Since the World Health Organization declared a pandemic on 11 March 2020, avoiding face-to-face activities and engaging in social distancing have become a part of everyday life. The pandemic has also induced changes in many countries’ educational environments as they began instituting online classes, including South Korea (hereinafter Korea), whose schools failed to begin the regular school year in March, for the first time in history. Despite this unprecedented situation, Korea is actively responding to social changes by offering a diverse school curriculum through online classes and developing new approaches to education. The changes required by the crisis may present an opportunity to adapt to the education needs of the incipient Fourth Industrial Revolution. In many studies preceding COVID-19, the possibility of online classes has been examined as a part of future education, in that online classes can provide highly efficient and diverse elective classes to self-directed students [1,2,3,4,5,6].

Physical education centers on physical activity and is clearly distinct from general knowledge-based subjects. Therefore, online physical education classes require special preparation and operation to communicate and practice the values of physical education well. Currently, as in-person school attendance and online classes are occurring in tandem around the world, there is a need to examine whether online physical education classes are being held and conveying the values of physical education appropriately. Prior studies on the efficiency and potential of online physical education classes, however, are limited [7,8,9]. One such study focused on physical education textbooks published by the University of North Carolina at Greensboro and suggested employing direct and indirect experiential activities in addition to physical activities [8]. It further proposed a teaching and learning strategy for the management of interaction and motivation, learner-centered classes, and the application of a blended learning strategy in middle school physical education classes [9]. However, most existing studies have only examined the efficiency of college classes, within limited areas; to the best of our knowledge, no studies have investigated the difficulties or efficient operation plans of middle and high school online physical education classes. Thus, there is a need to identify the existing practices of and best directions for future online physical education classes, both during and after the pandemic. This study identifies the difficulties of middle and high school online physical education classes and suggests ways to efficiently manage future online physical education classes. The results may serve as basic material to help revitalize online physical education classes in the future.

## 2. Materials and Methods

The study employed a qualitative case study method using phenomenological procedures to collect and analyze the data [10]. “Turning to the nature of lived experience” of research participants’ online physical education classes, the study explored the experience of conducting these classes, discussed and reflected on their efficient operation and difficulties experienced therein, and examined the data by “writing and rewriting”.

### 2.1. Participants

To find a generalized representation of middle (14–16 years old) and high (17–19 years old) school online physical education classes in Korea, the researcher selected six participants for this study, who were recommended by the Korea Ministry of Education and the Council for School Physical Education Promotion, which pursues the revitalization of physical education in Korea. Three participants were middle and high school physical education teachers who were experts in online physical education; the other three had worked to improve the three types of online classes offered by the Korea Ministry of Education. All participants provided informed consent to participate in the study, which was approved by the Korea Jeonbuk National University High School. Table 1 shows the characteristics of the research participants.

### 2.2. Data Collection

The collected data included material directly produced by the research participants and online videos of their physical education classes. In-depth individual and group interviews were conducted to examine experiences emerging in the participants’ journals. We examined the participants’ personal diaries and their online physical education class operations. Five in-depth individual participant interviews lasting 50–70 min were conducted between March and June 2020. The interviews began with participants describing individual operation plans and were centered on the operation of these cases. Five group interviews lasting 60–90 min were also conducted from April to June 2020, focused on difficulties that were encountered and overcome in the online physical education classes. The group interviews were comprised of open discussions among the research participants, which allowed collaborative and interpretive reflection within a seminar format.

### 2.3. Data Analysis and Research Authenticity

An inductive category analysis was employed, focusing on open coding, axial coding, and core coding [11]. The researcher worked to understand the overall flow and true meaning of the material through repeated reading. The meanings were classified and grouped by subject and analyzed through technical, reflective, and interpretive writing; then, the relationships between the essential elements of the results were identified to determine the overall structure. Finally, an iterative process of reinterpretation, modification, and integration was applied to ensure that the generated categories reflected the purpose of the study.

To enhance the validity of the study and test the consistency of the findings, a triangulation technique cross-verified data through an in-depth description from various angles using the collected data and the researcher’s notes. The derived results were reviewed by the participants to ensure that their meanings were accurately expressed. The quality of the study was ensured through continuous feedback from two qualitative research experts (Professor “S” of “J” University and Professor “L” of “S” University), who reviewed the entire study process.

## 3. Results

### 3.1. Difficulties in Running Online Middle and High School Physical Education Classes

#### 3.1.1. Conveying the Value of Physical Education

Difficulties in conveying the value of sports in online physical education classes remained in the modified technical practice. This value included maintaining health through physical activities, cultivating community consciousness through physical activities with friends, and developing sports etiquette through sports participation. Students engaged in online physical education classes often cannot secure enough space to effectively take part in physical activity and also have limited access to supplies and equipment needed to follow online physical education classes. Thus, the participants running the online physical education classes used supplies that were readily available at home, which necessarily reduced the physical education units that could be taught. This led to a shift in focus from competition, which is a major part of in-school physical education, to health and physical activity challenges in online instruction.

Teacher “A”: *In online physical education classes, students had to participate alone and use the supplies at home, so it was inevitable that classes were limited. However, it was easy for me to give feedback because I run a real-time interactive class and students practice it immediately in line with my fitness program*.

Teacher “C”: *Real-time interactive classes can be effectively used in a small class, but it seems inefficient in a class of about 30 students. Thus, I used lecture-type content to provide explanations and demonstrations, present assignments, and give feedback*.

Teacher “b”: *I run a content-oriented class, but I had doubts about whether the values of physical education that we wanted to deliver were being conveyed well, given the limited environment and the fact that students had to practice alone*.

Teacher “c”: *I agree. I had actually planned a class in the competition area, but I could only do classes in the health area. I was worried that the students would feel too complacent about physical education through such classes*.

Teacher “a”: *I had no choice but to run really monotonous classes like juggling and “challenging” stay-at-home challenges that could be done in students’ own houses*.

(From the first group interview).

In contrast to the general knowledge focus of core subject courses, physical education focuses on physical activity, an emotional domain. All participants had concerns about how to convey physical activities in online physical education classes and how to make the online physical education class a meaningful educational activity. In a study of physical activity limitation, Kim et al. [12] reported that various physical educational activities geared toward health should be included in an online class, as most participants, despite various ages and genders, had health problems.

It is possible that online physical education classes can be made more efficient if students receive feedback through viewing their own or their classmates’ actions. This is in contrast to face-to-face physical education classes, where students can immediately receive feedback on their motor skills or their success completing physical activities. In contrast, students cannot modify their own activities by viewing a video of them, so they receive limited feedback. Immediate feedback is needed to motivate students to learn and strengthen their active class attitude. The participants tried to provide feedback across time and space through online media; however, this was difficult, because basic rapport between the teacher and the students and among the students themselves was not able to develop well through the online approach. In addition, the lack of interaction between the teacher and students in online courses made it difficult to convey the value of physical education.

*There was an interaction between teachers and students when the teacher provided feedback by checking students’ online assignment performance. This interaction became an advantage of interactive physical education classes and assignment-oriented physical education classes. However, this was difficult because basic rapport was not developed through the online approach. In addition, the lack of interaction between the teacher and students in online courses made it difficult to convey the value of physical education*.

(From the in-depth interview of Teacher “a”).

Like the result of the in-depth interview with Teacher “a,” the interaction between the teacher and the student becomes an important factor for the realization of the value of physical education. This experience suggests that attempts to convey the value of physical education should be initiated later in the semester, after rapport has been developed between the teacher and their students and after the technical skills for various sports have been reviewed [13].

#### 3.1.2. Lack of Teacher Experience

Online physical education classes, instituted nearly worldwide during the 2020 pandemic, were a wholly new experience for both teachers and students. The sudden shift to online classes left teachers unprepared and struggling with unfamiliar teaching methods, forcing them to resort to trial-and-error approaches. Inadequate online teaching strategies and low teacher and student readiness for online classes made the transition difficult [14].

*I had to think about the content of physical education classes that I could do online with the start of online classes due to COVID-19, and about the content of the class that could be evaluated when students came to school later. The content of online physical education classes were selected based on individual sports that can be done while maintaining social distancing after school starts. However, as the use of various evaluations (individual evaluation, group evaluation, etc.) was limited due to restrictions on class activities by group, I was very worried about what to do*. (From the in-depth interview of Teacher “b”).

*The filming and production of online class materials by the physical education teacher himself took two to three times longer to prepare (e.g., production and editing) than the existing physical education classes. Even if various content (YouTube, Internet materials, etc.) was used, it took a lot of time and effort to search for videos and materials that matched the teaching content of the physical education teacher’s class*. (From the in-depth interview of Teacher “C”).

The participants’ principal concerns about running online physical education classes centered on the lack of efficient content and difficulties in using the content. They worried about the students’ ability to participate in sufficient physical activities given space restrictions and the online course content they created, and whether the course content was educationally meaningful. The availability of media to capture and edit various physical activity photos and videos was absolutely essential for online course preparation. The participants experienced considerable confusion in their initial attempts at online instruction, although the Ministry of Education and the municipal and provincial education offices provided guidance and training on operating online classes and copyright issues after the switch to online classes.

*I feel that it is more important than anything else for physical education teachers to develop their ability to efficiently use content in the areas where various aspects of physical activity are expressed and where the content of explanation, demonstration, and feedback is provided. This is an important point that I realized while lecturing in the content utilization training course due to the fact that physical education is unlike the general subjects. I believe that my experience in online physical education classes will definitely be an opportunity*. (From the research journal of Teacher “A”).

*The physical education teachers had to revise their education plans, courses, and evaluations several times in their online physical education classes. It is true that it is very confusing. I am going through a lot of difficulties because it is my first time using the content of online physical education classes and making evaluations*. (From the in-depth interview of Teacher “B”).

Physical education teachers who were familiar with online content could easily incorporate it. However, others had difficulties even with simple tasks, such as uploading lectures and linking videos from different sites. Those who developed their own lectures experienced difficulties preparing for online physical education classes, because they lacked the necessary equipment (cameras, microphones, laptops, etc.), had no access to software for editing images and coding video files, and/or lacked experience in using such software. To maximize the efficiency of online physical education classes, both teacher effort and collaboration with online experts were essential [7].

#### 3.1.3. Evaluation

The Ministry of Education presented guidelines for evaluating online classes [15], which specified that teachers were to refrain from conducting evaluations unless they could be done face-to-face and recommended conducting evaluations after the return to in-class instruction to the extent possible. Participants found it difficult to apply evaluations to online physical education classes. It seemed unreasonable to evaluate students on what they had learned in school following a long period of online classes—especially if these were conducted solely through lectures and assignments without the students actually performing and practicing the activities to be evaluated—particularly because the proportion of the evaluation based on physical activity was high, given the nature of the subject of physical education. This differs from general subject evaluations, where written examinations based on online course work can be administered after the return to in-school classes. Although students could submit physical education performance evaluations in the form of videos and written assignments, it would be very time-consuming for large schools to determine whether students had submitted the evaluation materials and then to actually evaluate those materials.

*In order to evaluate a gymnastics movement, I asked the students to take a picture of themselves doing the gymnastics movement and upload it. However, there were limits in uploading the entire gymnastic movement, and so the evaluation was made in partial movements. In addition, there was too much restriction in providing feedback and evaluation for all images*. (From the in-depth interview of Teacher “c”).

*It has been a while since online physical education classes started, but I don’t believe that the performance evaluation proposed by the Ministry of Education is a concrete plan yet. Evaluations must be done in terms of efficiency and expandability of online physical education classes*. (From the in-depth interview of Teacher “B”).

Teacher “c”, who had been conducting performance evaluations based on assignments, found it difficult to complete the evaluations, because performance assessment was not conducted in real time. In addition, she felt that the diversity and specificity of the evaluation was very poor because they were limited to evaluating individual activities through videos. Each study participant completed evaluations according to the type of online physical education classes they conducted, and all participants described encountering specific difficulties in completing the evaluations.

Teacher “A”: *It is very difficult to check the performance of what students practiced in real-time interactive classes*.

Teacher “a”: *The home training and yoga practice scenes were evaluated in real time, but the evaluation took too long*.

Teacher “B”: *The performance assignment was checked through simple quizzes and discussions during the content-oriented class, but there were many difficulties in evaluating the actual activities and conducting detailed evaluations*.

Teacher “b”: *I believe that the evaluation is essential for online physical education classes. For self-directed learning, the evaluation parts associated with the assignment should be presented in various forms*.

Teacher “C”: *Many teachers spend too much time giving feedback and evaluations in assignment-oriented classes. Systematic supplementation is needed online*.

Teacher “c”: *Since there is a very limited amount of information that can be recorded in the Student Record in the existing evaluation, a new evaluation method that can evaluate and record the learning process should be introduced*.

(Summary of the discussion on evaluations in the second and third group interviews).

In the second and third group interviews, participants discussed the difficulties of the evaluation and argued that evaluation concepts and practices for online physical education classes should be re-established based on the current evaluation results. They likewise argued that these concepts and practices should include measures that confirm whether students actively participated in the online physical education classes. In addition, physical activity content that can be viewed online needs to be expanded.

### 3.2. A Plan for the Efficient Operation of Middle and High School Online Physical Education Classes

#### 3.2.1. Content that Conveys the Value of Physical Education

Online physical education classes need to teach the value of physical activity as an important element of health [16]. However, before teaching students the value of physical education, teachers should focus on physical education concepts while preparing students to actively participate in the online class. Online physical education classes should teach students to subjectively develop future physical activity plans and self-directed competencies. Although the internet delivers classes without time and space constraints that nearly everyone can access, such classes are ineffective and inefficient if students do not actively and responsibly participate. In other words, the students’ attitude toward self-directed learning is an important factor in the efficient operation of online physical education classes. Therefore, teachers need to develop educational strategies for online classes that help students form a learning attitude. Engaging and motivating students to participate in physical activities can help convey the value of physical education [17].

*Teacher “b”: When conducting training for teachers, the issue was raised that no matter how much effort is made by the teacher to conduct a good class, it will be of no use if the students are not willing to listen. In such a case, the plan needs to be re-examined*.

*Teacher “C”: Yes, that is correct. If the online physical education class begins and no assignments are given, it would not be possible to check if the student is listening to the online class. Actually, some students do assignments without listening to assignments, which means you can set a group for the class and complete the group work outside of class. Thus, I have tried interactive classes among students to complete a set of assignments as a group*.

*Teacher “A”: That’s a good idea. Before discussing the value of physical education, it should be preceded by many educational devices and materials so that students can listen to online classes with an attitude toward self-directed learning*.

*Teacher “B”: Yes, I agree. The value of physical education should be naturally achieved in class, and a good class will be meaningless if the students do not have active learning attitudes*.

Teacher “c”: *Yes, I have tried to make changes in the existing physical education class by making students submit reports and videos based on their activities to make them actively participate in class*. (From the fourth group interview).

In the group interviews, participants discussed the buzz learning method as a way to increase student participation in online classes [18]. Changes are essential for developing and applying group assignments that encourage student participation to overcome the disadvantage of online physical education classes [18]. New assignment content needs to be developed in the future that will allow teachers to identify an individual student’s learning status, just as the research participants developed different educational strategies to increase the value of the class. Physical activity does not necessarily need to be central in the actual class to establish the value of physical education; Park et al. [19] reported that the establishment of the value of physical education based on various types of materials is necessary in online physical education classes, as various audiovisual aids and activity equipment are provided to support the positive health behavior of university students. There is a need to develop ways to link the emotional areas while expanding the cognitive and defining areas, which can be an advantage of online physical education classes.

*Teacher “b” made great efforts to motivate and interest students by using physical education textbooks to explain theoretical aspects and presenting images to help students understand the material. Indirect experience based on direct experience of physical activity and the value of physical education were delivered through intensive classes in cognitive areas using physical education textbooks*. (Analyzing the content of Teacher “b’s” online physical education class).

*I do not think that it is necessary to teach the value of physical education centered on physical activity. Rather, I think that by running this online physical education class, I was able to deliver the value of integrating various topics through theoretical classes in physical education textbooks. I tried to convey the value of physical education by using various video images, arguments, discussions, and reporting that were not well utilized in existing physical education classes*. (From the in-depth interview of Teacher “b”).

Online physical education classes are clearly different from traditional physical education classes. Participants made changes while running online physical education classes and conveyed the value of physical education in different ways.

#### 3.2.2. Efforts to Cultivate Teacher Expertise

Participants pointed out that one change driven by online physical education classes was the active progress made by physical education teachers through collaboration, which provided training and help to teachers who had difficulty creating content in the early stage of online classes. This collaboration naturally expanded as they produced class videos and shared ideas on assignment methods and structures and class content. This collaboration was driven by the power of collective intelligence within the physical education community and demonstrated a culture of sharing based on the autonomy of the Physical Education Research Society and networks among colleagues [20].

*Considering that this is my first online class this year, the most distinguishing feature is that there is a place where physical education teachers from a variety of schools share the materials, content, and concerns regarding online physical education classes. Would you say that we were tightly united in a crisis? It seems to have served as an opportunity for physical education teachers to reduce the trial-and-error and to develop better physical education classes*. (From the in-depth interview of Teacher “C”).

*The research participants’ videos showed that physical education teachers collaborated on making demonstrations and teaching, thereby producing more professional content by producing a joint video that fit the class subject*. (From the researcher’s journal).

The importance of the teacher learning community is reported in many studies on the development of teacher expertise [21,22,23]. Physical education class videos continue to be produced and teachers continue to cultivate their expertise as they develop and produce these class videos. Research participants continued to develop their expertise by searching for educational materials, including carefully examining materials from The Council School Physical Education Promotion and the Physical Education Research Society, while developing online physical education classes. They further developed their expertise by producing and editing their own videos. The results of their efforts provide a good example of how to effectively prepare for future physical education.

*I was at a loss when I first started preparing for online physical education classes, but I received a lot of help from the teachers at the Physical Education Research Society. In addition, it really helped me cultivate my expertise while reflecting on my class. It was also very helpful to be able to view the classes of other physical education teachers, which used to be hard to see before*. (From the in-depth interview of Teacher “a”).

*It was great to be able to look at the really valuable materials in The Council School Physical Education Promotion and the National Physical Education Teacher Group’s “katokbang”. It was good to see many physical education teachers collaborate and build their expertise in “an opportunity that lies in a crisis”. That is why I became confident in my class, too*. (From the in-depth interview of Teacher “c”).

Physical education teachers who strive to improve their expertise give students faith in the subject. Faith creates interdependence through communication between the teacher and the students and also acts as an “invisible bridge” in physical education classes [24]. Faith between the teacher and students can also be indirectly formed by the teacher’s demonstrating instructional content and expertise while running an online class. Efforts are needed to cultivate professional and practical knowledge suitable for online physical education classes through changes in teaching and learning methods, interaction with students, a broad understanding of the area, and expanded knowledge.

#### 3.2.3. Preparation for Improved Evaluations

Online physical education performance is difficult to evaluate. Traditional evaluations are extremely limited, including online and offline integrated evaluations, process-oriented evaluations, and physical activity-oriented evaluations. The research participants adapted their evaluation methods to determine whether the student achievement standards were met and whether advancement to the next class was appropriate.

*Teacher “A”: Teacher evaluation is conducted by looking directly at the student’s activities. Peer evaluation is conducted by students looking at one another*.

*Teacher “a”: Our evaluation method entails showing various videos that fit the topic of the class and talking about the feelings they elicit in real time*.

*Teacher “B”: There is no direct evaluation, and the achievement standards are reviewed by looking at the class and simply writing the overall content in the form of a report*.

*Teacher “b”: A self-assessment is conducted to determine whether the student has participated in class with an attitude toward self-directed learning, and whether the student has completed the assignments, but they are not reflected in the student’s score*.

*Teacher “C”: Evaluations cannot be made because it is an assignment-oriented class. Images of the student’s physical activity are used to deliver feedback through student self-assessment and teacher evaluation*.

*Teacher “c”: Based on the attached content of assignments carried out by the student, the course is recorded in the physical education section of the student’s Study Record*.

(Summary of evaluation discussion in the fourth and fifth group interviews).

One characteristic of online education is that students can develop unique thinking through learning activities that meet their needs and cultivate creativity through the process of thinking [25]. Evaluation methods need to be improved to capture the process of verbalizing students’ thoughts. It is necessary to conduct evaluations in the form of an inspection to understand the educational value of online physical education classes, much like the way in which the research participants expanded the evaluation to assess diagnosis, formation, and achievement in addition to performance.

*The above student faithfully carried out the assignments regarding national health gymnastics during online physical education classes, understood and analyzed teacher and peer evaluation feedback, and faithfully participated in the assignments*. (From the examples of Study Records by Teacher “c”).

*Teacher “A” evaluated interactive lessons in real time, but emphasis was placed on the students who delivered feedback and made corrections according to the feedback. In addition, a peer evaluation method was applied to the class in which feedback was provided by watching videos that in real-time interactive class, meaning other students watch the monitor video between students through informal evaluation*. (Analyzing the content of Teacher “A’s” online physical education class).

Research participants used informal evaluations to record student participation in the Study Record as a way to induce active participation. This was done while using the performance evaluation content required in physical education classes as a learning strategy. Evaluation of the online classes, which was conducted for the first time in 2020, is not yet concrete, and efficient evaluation methods and content should be examined in future studies.

## 4. Discussion

This study examined the difficulties teachers experienced in running online physical education classes following the start of online schooling in Korea in the context of COVID-19 and presented an efficient operation plan for future online physical education classes.

The difficulties of operating online middle and high school physical education classes included monotony related to limited environmental conditions and educational content, which ultimately decreased the effectiveness of conveying to students the value of physical education. It is necessary in this light to discuss the value of physical education during online classes. Second, physical education teachers across the country lacked expertise in employing online content and had to resort to trial-and-error methods. To address problems like these, we expect that effective content will develop in various directions due to the COVID-19 outbreak. Third, student evaluations conducted in accordance with the evaluation guidelines proposed by the Korea Ministry of Education were very limited, and a systematic evaluation was not possible because of the online nature of the classes. There is a possibility that a new evaluation method that can be operated effectively in online classes will need to be constructed.

In addition, to develop effective online physical education classes, strategic learning methods that incorporate online physical education characteristics are needed to help teachers communicate the value of physical education. In delivering the values of physical education, which is the goal of physical education in Korea, addressing the psychodynamic domain and affective domain, which are lacking in online classes, will certainly improve the efficiency of online physical education classes. Second, physical education teachers need to prepare for the future methodology of physical education and acquire professional practical knowledge through sharing online physical education content. This collaboration among physical education teachers is central and should incorporate expertise from the Korea Physical Education Research Society. Third, it is necessary for students to make an effort to actively participate in online physical education classes and record the process in their life record books through discussion of evaluation methods and methods suitable for an online physical education class.

In this study, the research participants did not have extensive experience in information and communication technology coming into the pandemic and the advent of online education, but they nevertheless actively participated in online physical education classes and played the role of representatives of Korea, making the active efforts required by the times. Finally, the need is apparent to explore various cases of online physical education, teachers’ and students’ experiences, and their meaning, to improve the generalizability of the lessons learned.

## 5. Conclusions

The study findings had several implications. First, it is necessary to study the state of different countries’ experiences in online instruction physical education instruction, comparing and analyzing how online physical education classes are conducted worldwide. Accordingly, there is a need to review and systematize approaches to online physical education classes that highlight each country’s cultural and educational characteristics and to examine the effectiveness of online physical education classes as a whole. Second, there is a need to explore the potential of online physical education classes linked to face-to-face physical education classes to examine their respective effectiveness and potential possibilities in light of physical education teachers’ increased expertise gained through their operation of online physical education classes. Third, future studies should establish a theoretical framework for online physical education classes by examining the educational value of modifying existing pedagogical methods, content, evaluations, and so on to more effectively teach online physical education classes. Fourth, future studies should also examine the efficiency and affordances of different online platforms employed by physical education teachers and evaluate their generalizability across actual school sites, especially as novel tools are developed.

## Figures and Tables

**Table 1:** Participant characteristics.

Online Physical Education Class Type	School Classes	Role	Gender	Participant	Research Participant Characteristics
Interactive PE Class	“N” Middle School (6 classes)	Teacher	Male	A	As a physical education teacher at “S” Middle School in the 7th year of his educational career, he runs a “Physical Enhancement Program”, an interactive PE class of about 20 students, utilizing Zoom. He is a training instructor for online PE content for physical education teachers nationwide and has a good understanding of the pros and cons of interactive PE classes.
	“I” High School (9 Classes)	Teacher	Female	a	As a physical education teacher for “I” High School in the 20th year of her educational career, she runs a “home training and yoga program” using Microsoft Teams, for a class of 15. While operating interactive teacher/student physical education classes, she tries to motivate student participation by using various video content and constantly strives for immediate feedback and interaction with students by asking questions via video.
Content-oriented physical education class	“J” Middle School (32 classes)	Teacher	Female	B	As a physical education teacher for “J” Middle School in the 11th year of her educational career, she runs a content-oriented physical education class using PPT and Open Broadcaster Software (OBS Studio) programs for a class of 30. She switched to a content-oriented physical education class after initially running an interactive PE class, in which many students found it difficult to participate.
	“J” High School (24 classes)	Teacher	Male	b	As a physical education teacher at “J” High School in the 15th year of his educational career, he runs a content-oriented physical education class using YouTube and videos he has produced for a class of 30. He runs a class that combines theory and practice using physical education textbooks. He also works as a lecturer for the J-region Physical Education Research Association.
Assignment-oriented physical education class	“H” Middle School (23 classes)	Teacher	Male	C	As a physical education teacher at “H” High School in the 23rd year of his educational career, he runs an assignment-oriented physical education class using basic lecture-type content for a class of over 30. In addition to physical activity assignments, he offers online group learning assignments to students and provides feedback during class. Currently, he works as a lecturer in the operation of assignment-oriented physical education classes nationwide.
	“G” High School (30 classes)	Teacher	Female	c	As a physical education teacher at “G” High School in the 4th year of her educational career, she runs an assignment-oriented physical education class for 30 students. The class is interactive and includes feedback from the teacher and focuses on “National Health Gymnastics” and “Creative Gymnastics” developed and practiced by students. The class uses Google Classroom and is equipped with assignment videos and explanations.

PE, physical education.

## References

[1] Blaine A.M. (2019). Interaction and presence in the virtual classroom: An analysis of the perceptions of students and teachers in online and blended Advanced Placement courses. Comput. Educ..

[2] Son C.H., Kang S.G., Ha S.J. (2016). Study on application of online instruction for enhancing rights for learning: Focusing on high schools. J. Creat. Inf. Cult..

[3] Lee E.J. (2010). A study on college students‘ perception on convenience in online courses. J. Educ. Inf. Media.

[4] Lee N.H., Jung H.R. (2018). The effects of the flipped learning sensory integration therapy class using online learning platform on learning participation. Korean Enterainment Ind. Assoc..

[5] Yong N. (2020). On-line classroom visual tracking and quality evaluation by an advanced feature mining technique. Signal Process. Image Commun..

[6] Jeong Y.S. (2014). Providing high school students with online instruction for optional curriculum. Korea Contents Soc..

[7] Lm H.J., Kim S.J. (2007). Development and application of e-learning contents to pre-service physical education teacher education. Korean J. Sport Pedagog..

[8] Lyu M.J. (2011). A case study on structure and possibility of online courses in physical education. J. Res. Curric. Instr..

[9] Hong S.H. (2006). A study on teaching and learning plan of physical education in middle school using blended learning strategy linked. Korean J. Phys. Educ..

[10] Van Manen M. (1990). Researching Liver Experience.

[11] Strauss A., Corbin J. (1998). Basics of Qualitative Research: Grounded Theory Procedures and Techniques.

[12] Kim S.E., Lee Y.S., Lee J.Y. (2020). Differences in causes of activity limitation by sex and age. J. Men’s Health.

[13] Guan B. (2012). Determination of China’s online physical education object. Procedia Eng..

[14] Do J.W. (2020). An investigation of design constraints in the process of converting face-to-face course into online course. J. Educ. Cult..

[15] Korea Ministry of Education (2020). Guidelines for Attending, Evaluating and Recording Remote Classes to Respond to Corona 19.

[16] Marilyn M.B., Jacalyn L.L., Joyce M.H., Connie B.C. (2006). Instructional Strategies for Secondary School Physical Education.

[17] Ahn Y.O. (2019). Inquiry on justification of the physical education on the view of value theory. Korean Soc. Elem. Phys. Educ..

[18] Lee Y.J., Gwak B.C. The need of buzz learning in real-time distance education. Proceedings of the Korean Society of Computer Information Conference.

[19] Park S.U., Ahn H., So W.Y. (2020). Developing a model of health behavior intentions and actual health behaviors of Korean male university students. J. Men’s Health.

[20] Jeong H.C., Kim D.J. (2018). Exploration of culture and meaning of participation in secondary physical education study group. J. Educ. Cult..

[21] Yoon K.J., Lee G.S., Lee C.H. (2018). A case study of using an online cafe in a physical education teacher learning community. Korean J. Sport Pedagog..

[22] Jo K.H., Lee O.S. (2016). An investigation of the impacts and factors influencing elementary teachers’ participation in a physical education teacher learning community. Korean J. Phys. Educ..

[23] Vangrieken K., Meredith C., Packer T., Kyndt E. (2017). Teacher communities as a context for professional development: A systematic review. Teach. Teach. Educ..

[24] Jeong H.C., Kim D.J. (2020). A self-study on the management experience of learner-centered physical education. Korean J. Sport Pedagog..

[25] Berge Z. (1997). Characteristics of online teaching in post-secondary, formal education. Educ. Technol..

